# Dissociation between skin test reactivity and anti-aeroallergen IgE: Determinants among urban Brazilian children

**DOI:** 10.1371/journal.pone.0174089

**Published:** 2017-03-28

**Authors:** Neuza M. Alcantara-Neves, Rafael V. Veiga, João C. M. Ponte, Sérgio S. da Cunha, Silvia M. Simões, Álvaro A. Cruz, Maria Yazdanbakhsh, Sheila M. Matos, Thiago Magalhães Silva, Camila A. Figueiredo, Lain C. Pontes-de-Carvalho, Laura C. Rodrigues, Rosemeire L. Fiaccone, Philip J. Cooper, Maurício L. Barreto

**Affiliations:** 1 Instituto de Ciências da Saúde, Universidade Federal da Bahia, Salvador, Bahia, Brazil; 2 Universidade Federal de Juiz de Fora, Juiz de Fora, Minas Gerais, Brazil; 3 Departamento Medicina Social, Universidade Federal de Pernambuco, Recife, Pernambuco, Brazil; 4 Departamento de Pediatria, Universidade Federal de Sergipe, Aracajú, Sergipe, Brazil; 5 ProAR- Núcleo de Excelência em Asma, Universidade Federal da Bahia, Salvador, Bahia, Brazil; 6 Department of Parasitology, Leiden University Medical Center, Leiden, The Netherlands; 7 Instituto de Saúde Coletiva, Universidade Federal da Bahia, Salvador, Bahia, Brazil; 8 Departamento de Ciências Biológicas, Universidade Estadual do Sudoeste da Bahia, Vitória da Conquista, Bahia, Brazil; 9 Centro de Pesquisas Gonçalo Moniz, Fundação Oswaldo Cruz, Salvador, Bahia, Brazil; 10 Institute of Infection and Immunity, St George’s University of London, London, United Kingdom; 11 Departamento de Estatística, Instituto de Matemática, Universidade Federal da Bahia, Salvador, Bahia, Brazil; 12 Faculty of Facultad de Ciencias Medicas, de la Salud y la Vida, Universidad Internacional del Ecuador, Quito, Ecuador; 13 Institute of Infection and Immunity, St George’s University of London, London, United Kingdom; Chang Gung University, TAIWAN

## Abstract

**Background:**

The dissociation between specific IgE and skin prick test reactivity to aeroallergens, a common finding in populations living in low and middle-income countries, has important implications for the diagnosis and treatment of allergic diseases. Few studies have investigated the determinants of this dissociation. In the present study, we explored potential factors explaining this dissociation in children living in an urban area of Northeast Brazil, focusing in particular on factors associated with poor hygiene.

**Methods:**

Of 1445 children from low income communities, investigated for risk factors of allergies, we studied 481 with specific IgE antibodies to any of *Blomia tropicalis*, *Dermatophagoides pteronyssinus*, *Periplaneta americana* and *Blatella germanica* allergens. Data on demographic, environmental and social exposures were collected by questionnaire; serum IgG and stool examinations were done to detect current or past infections with viral, bacterial, protozoan and intestinal helminth pathogens. We measured atopy by skin prick testing (SPT) and specific IgE (sIgE) to aerollergens in serum (by ImmunoCAP). SIgE reactivity to *B*. *tropicalis* extract depleted of carbohydrates was measured by an in-house ELISA. Total IgE was measured by in house capture ELISA. SNPs were typed using Illumina Omni 2.5.

**Results:**

Negative skin prick tests in the presence of specific IgE antibodies were frequent. Factors independently associated with a reduced frequency of positive skin prick tests were large number of siblings, the presence of IgG to herpes simplex virus, *Ascaris lumbricoides* and *Trichuris trichiura* infections, living in neighborhoods with infrequent garbage collection, presence of rodents and cats in the household and sIgE reactivity to glycosylated *B*. *tropicalis* allergens. Also, SNP on *IGHE* (rs61737468) was negatively associated with SPT reactivity.

**Conclusions:**

A variety of factors were found to be associated with decreased frequency of SPT such as unhygienic living conditions, infections, total IgE, IgE response to glycosylated allergens and genetic polymorphisms, indicating that multiple mechanisms may be involved. Our data, showing that exposures to an unhygienic environment and childhood infections modulate immediate allergen skin test reactivity, provide support for the “hygiene hypothesis”.

## Background

Atopy can be defined by either a positive skin prick test (SPT) or the presence of allergen-specific IgE in serum (sIgE) and, generally in high-income countries, both measurements tend to be consistent in the same individual [1]. It is expected, therefore, that an individual with a detectable sIgE would also have a positive SPT to the same allergen, and vice-versa, but for practical reasons SPT has been more frequently used in clinical practice and research. However, there is growing evidence for a dissociation between SPT positivity and sIgE, particularly in marginalized populations living in low and middle-income countries. The dissociation has been observed either as a negative SPT in subjects with detectable sIgE or, less frequently, as an absence of sIgE in a SPT-positive individual. The ISAAC phase II study involving large samples from affluent and non-affluent populations observed a dissociation between detectable sIgE and negative SPT in four non-affluent study centers ranging 31.1–78.9% [1]. Other studies, conducted in rural children of Europe [2] or Africa [3,4], have shown also large proportions of children with positive sIgE to be SPT negative for the same allergens. A possible explanation for this dissociation is the down-regulation of allergic effector responses in skin, such that individuals with measurable levels of sIgE fail to develop immediate hypersensitivity responses. Some environmental exposures, in particular helminths [3–5] and other childhood infections [2–6], have been associated with a suppression of *in vivo* skin immediate (Type I) hypersensitivity responses.

IgE reactive to carbohydrate epitopes present in both helminth antigens and allergens have been described–such IgE seems to have a lower affinity for the high affinity IgE receptor (FCɛRI) present in the cells that mediate Type I hypersensitivity reactions [7] such as mast cells and basophils, leading to a failure of their activation and degranulation. In addition, polymorphisms (SNPs) of IgE and FCɛRI-encoding genes may regulate IgE levels [8] or interfere with mast cell degranulation either by altering the IgE molecule itself or its affinity for FCɛRI.

To examine the hypothesis that poor hygiene causes the dissociation between SPT and sIgE in among non-affluent populations living in poor regions of low and middle-income countries, we studied children living in poor neighborhoods in a large Brazilian city. We analyzed data associated with poor environmental hygiene and childhood infections and also genetic factors to identify potential determinants of a reduced SPT reactivity among children with detectable levels of sIgE for the same allergens.

## Material and methods

### Study population and data collection

The study was a *post hoc* analysis of data collected during a survey of 1,445 children aged 4–11 years and living in 24 poor neighborhoods in the city of Salvador, Northeast Brazil. The study was done in 2005 as part of a cohort study to investigate risk factors for asthma and allergy and is described in detail elsewhere [9]. Neighborhoods and children were selected for a previous study to measure the impact of sanitation on diarrhea [10]. Here, we have analyzed data from those children with detectable sIgE (≥0.70 kU/L) to at least one of four tested allergens and for whom complete data for other relevant variables were available. Data on asthma symptoms were collected using a Portuguese-adapted ISAAC Phase II questionnaire. The following measurements were performed for each child: anthropometric measurements, SPT testing and serum sIgE to four aeroallergens, circulating IgG against six pathogens, stool examination for intestinal helminthic infections, and dust samples from children´s beds to measure relevant aeroallergens. In a subgroup of this population, sera from *Blomia tropicalis*-sensitized children were tested for IgE to *B*. *tropicalis* extract that had either been treated or not treated with methaperiodate to deplete carbohydrates. Data were collected also on the presence of cockroaches, rodents, cats or dogs in the household, and the proportion of households linked to a sewage system and with daily garbage collection. Children were genotyped for polymorphisms on *FCER1A*, *IGHE* and ancestry informative markers (AIMs) as described previously [11].

### Allergen SPTs

SPTs were done by two trained technicians using a standardized protocol using extracts of *D*. *pteronyssinus*, *B*. *tropicalis*, *B*. *germanica*, *P*. *americana*, dog and cat epithelia, and a fungal allergen mix (ALK-Abelló, São Paulo, Brazil). Extracts, saline and histamine controls were pricked onto the forearm skin using a disposable lancet (ALK-lancet®; ALK-Abelló, São Paulo, Brazil). Reactions were read after 15 minutes and a reaction was considered positive if the mean diameter of the wheal was 3 mm or larger than the saline control. Frequencies of positive skin test reactions to dog and cat epithelia and a fungal allergen mix were low (<4%) and were excluded from further analysis.

### Detection of intestinal helminth ova in fecal samples

Two faecal samples were collected two days apart and analyzed using the Hoffman sedimentation method and Kato-Katz thick-smear technique [12] for the presence of helminth parasites (*Trichuris trichiura*, *Ascaris lumbricoides*, hookworms and *Schistosoma mansoni*). Hookworms and *S*. *mansoni* infections were rare (<1%) and were not considered further.

### Dust

Dust was collected using a 1,200W vacuum cleaner (Electrolux, São Paulo, Brazil) with a 25 μm polystyrene filter from an area of 1m^2^ from the upper part of the child’s mattress for 2 minutes. The concentrations of the allergens Blo t 5, Der p 1, Bla g 2, Can f 1 and Fel d 1 were measured using commercial kits (Indoor Biotechnologies, Virginia, USA) following the manufacturer’s instructions.

### Serum immunoassay for IgG to bacteria, protozoa, and viruses

Serum IgG antibodies to *Helicobacter pylori*, *Toxoplasma gondii*, herpes simplex virus (HSV), herpes zoster virus (HZV), Epstein-Barr virus (EBV) were measured using commercial ELISA kits (Diamedix, Miami, Florida, USA; Adaltis, Toronto, Canada). For hepatitis A virus (HAV), kits from ADALTIS were used (Toronto, Canada). Assays were performed following the manufacturers’ instructions.

### Serum immunoassays for detection of total and allergen specific IgE

Total IgE was measured by in-house capture ELISA as described [13]. For the present analysis total IgE results were stratified into tertiles, corresponding to the following concentrations: <0.04, 0.04 - <0.1, and 0.1> ng/mL for 1^st^, 2^nd^, and 3^rd^ tertiles, respectively.

sIgE to *Dermatophagoides pteronyssinus*, *Blomia tropicalis*, *Blatella germanica* and *Periplaneta americana* was measured in sera using the Immunocap System (Pharmacia AB, Uppsala, Sweden) according to the manufacturer’s instructions. Sera with ≥0.70 kU IgE to any of the four allergens were considered positive. This cut-off we have used previously [13–15] to minimize positive reactions associated with high levels of low-affinity and cross-reactive IgE in helminth-infected populations.

### Detection of anti-*B*. *tropicalis* IgE using total or carbohydrate depleted extract

An in-house indirect ELISA was done to measure IgE to *B*. *tropicalis* before and after treatment with sodium metaperiodate (VETEC, Rio de Janeiro, Brazil). Briefly, delipidated *B*. *tropicalis* were added to microassay plates, some of which were treated with 10 mM sodium metaperiodate in 50 mM acetate buffer, pH 4.5, for 1 hour at room temperature in the dark, so that carbohydrates were oxidized to aldehyde. The assays was run as described previously [16]. Percentage of carbohydrate determinant-reactive anti-*B*. *tropicalis* IgE (BtE), in relation to total anti-*B*. *tropicalis* IgE (BtE), was calculated in accordance with the formula: % of carbohydrate-reactive to BtE = [1.0 - (mean OD of methaperiodate/boro-hydride-treated wells/mean OD of untreated wells).

### DNA extraction and genotyping

DNA was extracted from peripheral blood using a commercial kit (Gentra® Puregene® Blood Kit (Qiagen). Subjects were genotyped using the 2.5 HumanOmni Beadchip from Illumina (San Diego, California, USA) for ten single nucleotide polymorphisms (SNPs) (rs2494262, rs2511214, rs2427837, rs2247584, rs16841987, rs2427825, rs7548864, rs2427827, rs12119226, rs2252226) on *FCER1A* and three SNPs (rs61737468, rs12884681, rs74091262) on *IGHE*. Individual genetic ancestry estimates were based on 370,539 SNPs shared by samples from the HapMap Project, the Human Genome Diversity Project (HGDP) in the SCAALA population. We used the ADMIXTURE software [17] to estimate the contribution from Africans, Europeans and Native Americans to the SCAALA individuals. All analyzed SNPs were in Hardy-Weinberg equilibrium. Non-template negative controls and genotyping-positive controls were included in each genotyping plate. Automatic calling was performed with a quality value above 99%.

### Statistical analysis

The aim of the analysis was to evaluate the effects of a number of exposures on frequency of SPT positivity among children with detectable sIgE. Analysis for all exposures was performed using a parsimonious procedure. First, we assessed the association between each exposure in isolation and SPT reactivity among children with detectable sIgE by univariate logistic regression. Second, we built a multivariable model that included all potential associations with P<0.20 from univariate analyses. Using backwards stepwise selection and likelihood ratio tests, all environmental and infectious exposures variable with P<0.10 were left in the model. A *priori* confounders of age, gender and parental asthma were kept in the multivariate model. Initial analyses were done using logistic regression models with random effects to adjust for neighbourhood clustering, but given that standard errors without controlling for clustering gave similar results to ‘unclustered’ models, we present here only results of the latter. The following variables were used in the analysis: excreta disposal classified as “good” (sewage system), “intermediate” (septic tank) and “bad” (hole in the ground or open air); number of other children living at home; presence of cats or dog; infestation of households by cockroaches and rodents; infections with *T*. *trichiura* and *A*. *lumbricoides;* positive IgG to *T*. *gon*dii, *H*. *pylori*, herpes simplex, varicella zoster, and Epstein-Barr viruses; and two neighborhood-level variables (the proportion of neighbourhoods linked to a sewage system and the proportion of neighbourhoods with daily garbage collection). Differences in sIgE levels from the indirect ELISA between SPT positives and negatives were evaluated using Mann-Whitney test while those between levels of anti-*B*. *tropicalis* IgE for glycosylated versus non-glycosylated extracts were done (only samples positive to the raw extract using a cut-off optical density of 0.250) using the Wilcoxon signed rank test for matched pairs. Genetic data were analyzed using logistic regression adjusted for sex, age, helminth infection and individual genetic ancestry markers using PLINK software. Analyses were done using STATA (version 8, StataCorp, TX, USA,) and SPSS (version 16, SPSS, Inc, Chicago, Ill, US.).

### Ethical considerations

Ethical approval was obtained from the Brazilian National Ethical Committee. Written, informed consent detailing all procedures to be carried out on the children was signed by the child’s parent or legal guardian.

## Results

1,355 of 1,445 children enrolled had both SPT and sIgE measured. Detection of serum sIgE equal or above 0.70 kU/L for at least one of four measured allergens (*D*. *pteronyssinus*, *B*. *tropicalis*, *B*. *germanica and P*. *americana*) or a positive SPT for at least one of the same four allergens was observed in 510 (37.6%) and 401 (29.4%) of the children, respectively. Among all tested children, 2.7%, 2.4%, 4.3% and 8.1%, had positive SPT but were negative for sIgE anti-*B*. *tropicalis D*. *pteronyssinus B*. *germanica* and *P*. *americana* respectively (data not shown).

The present analysis was restricted to 481 children with sIgE (≥0.70kU/L) to at least one of the four aeroallergens. No statistically significant differences were seen for outcomes and variables between children included and children excluded from the analysis (data not shown).

Table 1 shows the frequency of SPT positivity in children with a sIgE ≥ 0.70kU/L for at least one allergen and for each individual allergen. Only 65.6% of children with detectable sIgE for at least one of the four specific allergens had a positive SPT positive for at least one of the same allergens. When individual allergens were considered, frequencies of positive SPTs varied from 74.2% (*P*. *americana)* to 33.5% (*B*. *germanica)*.

**Table 1:** Frequencies of positive skin prick tests (SPT) in 481 children with aeroallergen-specific IgE (sIgE).

Specific IgE	N	STP ≥ 3mm	
positivity (≥0.70 kU/L)		N	%
At least one allergen	491	322	65.6
*B*. *tropicalis*	437	244	55.8
*D*. *pteronyssinus*	273	168	61.5
*B*. *germanica*	182	61	33.5
*P*. *americana*	120	89	74.2

Table 2 shows crude and adjusted analyses for statistically significant associations between SPT reactivity to at least one allergen or *B*. *tropicalis* or *D*. *pteronyssinus* allergens and demographic, environmental, social variables and infection markers among children with positive sIgE. Associations for non-significant variables are provided in S1 Table (presence of cockroach, dog at home; presence of Blo t 5, Der p 1, Bla g 2, Can f 1 allergens in bed dust samples; seropositivity to Hepatitis A, *Herpes zoster* viruses, *Toxoplama gondii* and *Helicobacter pylori; A*. *lumbricoides and T*. *trichiura* and total serum IgE for one, two or all tested outcomes). In multivariable analyses (Table 2), we observed negative associations between SPT to at least one allergen and number of siblings, intestinal helminth infections, seropositivity to Epstein-Barr and Herpes simplex viruses, rodent infestation, household cats, and low neighborhood coverage for garbage collection. SPT to *B*. *tropicalis* was negatively associated with number of siblings and household cats. SPT to *D*. *pteronyssinus* allergens was negatively associated with low neighborhood coverage for garbage collection and with *H*. *simplex* infection. *P*. *americana* or *B*. *germanica* skin reactivity was not associated with any of the variables among children with sIgE for each of these allergens (data not shown).

**Table 2:** Crude and adjusted odds-ratio (OR) of statistically significant associations of possible determinants with skin prick test (SPT) to at least one allergen or *B*. *tropicalis* or *D*. *pteronyssinus* in 481 children with specific IgE to the respective allergens.

Possible determinants	Positive SPT for at least one allergen in children with specific IgE		
	N(%)	Crude	Adjusted
		OR(95%CI)	OR(95% CI)
**No. of siblings**			
0–1	211 (73.3)	1	1
> = 2	105 (54.4)	**0.44 [0.30–0.64]**	**0.54 [0.35–0.81]**
*Intestinal helminth infection			
No	283 (69.9)	**1**	**1**
Yes	33 (43.4)	**0.44 [0.28–0.68]**	**0.55 [0.34–0.89]**
**Rodent infestation in household**			
No	158 (72.5)	**1**	**1**
Yes	158 (60.1)	**0.57 [0.39–0.84]**	**0.61 [0.40–0.93]**
**Cat in household**			
No	296 (68.1)	**1**	**1**
Yes	20 (43.5)	**0.36 [0.19–0.67]**	**0.27 [0.13–0.54]**
**Epstein-Barr virus**			
No	50(78.1)	1	**1**
Yes	269(63.4)	**0.48 [0.26–0.91]**	**0.49 [0.26–0.93]**
**Herpes simplex virus**			
No	171(73.1)	**1**	**1**
Yes	151(58.8)	**0.52 [0.36–0.77]**	**0.50 [0.34–0.74]**
**% of neighborhood with daily garbage collection**			
> = 66%	109 (71.7)	1	1
33% - 66%	39 (76.5)	1.28 [0.61–2.68]	1.67 [0.75–3.72]
0–33%	168 (60.4)	**0.60 [0.39–0.92]**	**0.56 [0.35–0.89]**
**Possible determinants**	**Positive SPT to *B*. *tropicalis* in children with specific IgE**		
**Number of siblings**			
0–1	164 (62.1)	1	1
> = 2	80 (46.2)	**0.52 [0.36–0.77]**	**0.58 [0.38–0.88]**
*Intestinal helminth infections			
No	217 (58.7)	1	1
Yes	27 (40.3)	**0.63 [0.39–0.99]**	0.80 [0.49–1.31]
**Rodent infestation in household**			
No	126 (63.0)	1	1
Yes	118 (49.8)	**0.58 [0.40–0.85]**	**0.58 [0.39–0.87]**
**Cat in household**			
No	231 (58.3)	1	1
Yes	13 (31.7)	**0.33 [0.17–0.66]**	**0.27 [0.13–0.57]**
**Possible determinants**	**Positive SPT to *D*. *pteronyssinus* in children with specific IgE**		
**% of neighborhood with daily garbage collection**			
> = 66%	56 (66.7)	1	1
33% - 66%	24 (70.6)	0.70 [0.36–1.36]	0.75 [0.37–1.56]
0–33%	88 (56.8)	**0.59 [0.39–0.91]**	**0.55 [0.35–0.87]**
***Herpes simplex* virus infection**			
No	94 (68.6)	1	1
Yes	74(54.4)	**0.56 [0.33–0.89]**	**0.54 [0.32–0.91]**

All adjusted ORs were adjusted for a priori confounders (age, gender and parenteral asthma), and *Ascaris lumbricoides* and *Trichuris trichiura* infections.

*Ascaris lumbricoides and *Trichuris trichiura* infections.

**Fig 1:**
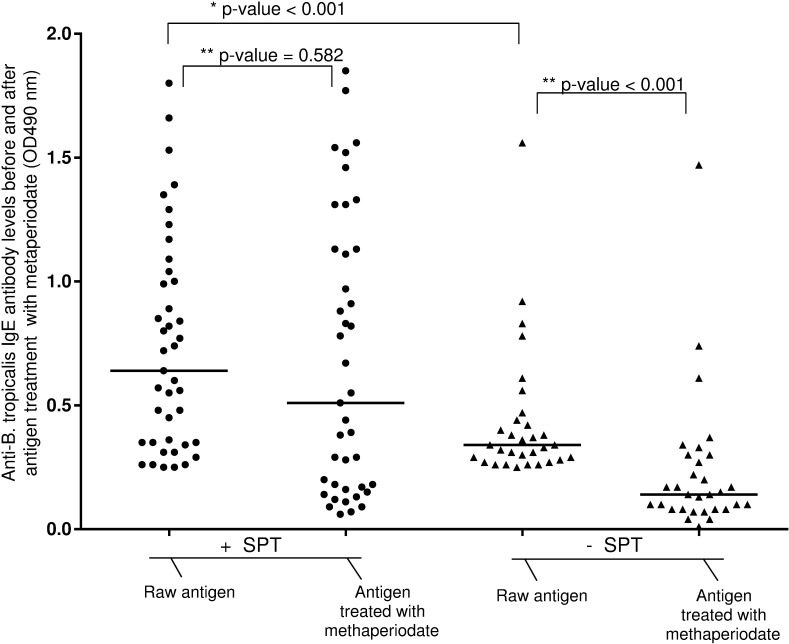
Anti-*Blomia tropicalis* IgE levels in serum samples assayed using as antigens *B*. *tropicalis* extract depleted or not of carbohydrate. Anti-*Blomia tropicalis* IgE levels were measured by indirect in-house ELISA in individuals positive for this antibody in IMMUNOCAP and were divided in two groups: SPT negative or positive for the same allergen. Antigen was treated with methaperiodate and the sera were assayed against raw and methaperiodate treated antigens. *Difference of sIgE results between SPT positive and SPT negative children (Mann–Whitney test). **Difference of sIgE results before and after antigen treatment with methaperiodate (Wilcoxon test).

Fig 1 shows that in general, children with positive SPT to *B*. *tropicalis* had higher levels of sIgE compared with SPT negatives (untreated, p<0.001). There was a small non-significant decrease in sIgE levels after methaperiodate treatment among SPT positives. However, in the case of SPT negatives, methaperiodate treatment caused a significant reduction in sIgE levels (p<0.001).

Table 3 shows the association between *FCER1A* and *IGHE* polymorphisms and SPT. Only one SNP (rs61737468), the T allele, on *IGHE* was associated with reduced SPT reactivity (OR: 0.29; 95% CI: 0.15–0.58; p<0.001). None of the SNPs on *FCER1A* were associated with SPT.

**Table 3:** Associations between *FCER1A* and *IGHE* polymorphisms withskin prick test (SPT) reactivity to at least one allergen among children with allergen-serum Ig.

Gene Polymorphisms	Alleles	MAF	OR^a^	95% CI	*P* value
***FCER1A***					
rs2494262	C/A	A	1.10	0.76–1.59	0.593
rs2511214	G/T	T	1.10	0.77–1.58	0.584
rs2427837	G/A	A	1.33	0.80–2.22	0.268
rs2247584	T/G	G	1.10	0.75–1.62	0.608
rs16841987	G/A	A	0.92	0.59–1.42	0.706
rs2427825	C/T	T	0.97	0.66–1.44	0.909
rs7548864	G/A	A	1.20	0.82–1.76	0.336
rs2427827	C/T	T	0.83	0.59–1.16	0.291
rs12119226	C/T	T	1.07	0.62–1.84	0.805
rs2252226	T/C	C	0.84	0.60–1.18	0.329
***IGHE***					
rs61737468	C/T	T	**0.29**	**0.14–0.58**	**<0.001**
rs12884681	A/G	G	0.94	0.67–1.33	0.748
rs74091262	C/T	T	0.65	0.33–1.28	0.271

We did not observe associations between levels of total IgE and SPT among children with sIgE either for any of the 4 aeroallergens tested or for specific aeroallergens (S1 Table). Then we analyzed also the role of total IgE as an effect modifier of the association between sIgE and SPT in the full study population of 1,355 individuals for which we had data (S2 Table). We observed that the association between sIgE and SPT was smaller in children with total IgE in the 3^rd^ tertile (representing levels >0.1 ng/mL) compared to those with IgE in the 2^nd^ tertile (P = 0.040).

## Discussion

In the present study, we explored potential factors that might explain the dissociation between SPT and sIgE to the same allergen that has been observed in marginalized populations living in low and middle-income countries (LMICs) such as Brazil. We analyzed data from children living in poor neighborhoods in the city of Salvador in a tropical coastal region of Brazil. Because previous studies have showed a low prevalence of SPT reactivity among children with childhood infections [4,14] we focused in particular on childhood infections, and factors associated with poorly hygienic living conditions. We observed statistically significant inverse associations between SPT reactivity and living in an unhygienic environment (i.e. greater number of siblings, intestinal helminth infections, having household cat and rodent infestations, and infrequent garbage collection) among children with aeroallergen-specific IgE (sIgE) indicating that such an environment may be an important determinant of this dissociation. The observation that distinct factors affected the SPT-sIgE association for *D*. *pteronyssinus* and *B*. *tropicalis* supports the idea that the mechanisms linking IgE with SPT for these two mites species may be distinct [18,19], although power for the analysis of individual aeroallergens was limited.

Other studies conducted in LMICs have shown dissociations between the presence of aeroallergen-specific IgE in serum and evidence of *in vivo* IgE-mediated skin hypersensitivity to the same allergens measured by SPT reactivity [2–4,20]. The environmental factors that may contribute to this effect were not clear [21,22]. Studies have shown a reduced prevalence of atopy measured either as SPT or sIgE among children with fecal-oral or food-borne infections such those caused by hepatitis A virus, *H*. *pylori*, *T*. *gondii* [6], non-typhoidal *Salmonella* [23] and helminths [24]. No previous study has investigated the effects of a wide range of social, environmental and infectious exposures on SPT reactivity among individuals with sIgE for the same allergens as we have done here. Consistent data from other studies include: (i) less SPT reactivity in Estonian compared to Swedish children despite similar frequencies of sIgE in both populations [2]; (ii) large dissociations between SPT reactivity and sIgE among children with helminth infections [4,5].

Possible explanations for the down-modulation of skin sensitivity to allergens are (i) induction of mechanisms regulating allergic effector responses [3,5,24], thus suppressing immediate skin hypersensitivity or (ii) differences in the affinity of IgE antibodies [25]. Chronic helminth infections have been shown to modulate the host immune system, and may have an important role in this immunomodulatory process [24]. Several studies have reported a reduced prevalence of SPT among children infected with systemic or intestinal helminths [26,27], but without reference to sIgE levels. We have reported previously in this population that *T*. *trichiura* infection early in life was associated with a reduced prevalence of SPT later in childhood [14]. Furthermore, we have shown that *Toxocara* spp. seropositivity acts as an effect modifier of the association between SPT and sIgE [15]. The data from the present study indicate that current *A*. *lumbricoides* or *T*. *trichiura* infections, measured by the presence of parasite eggs in stool, are associated with a reduced SPT positivity to any allergen among children with sIgE. These findings may reflect possible IgE cross-reactivity between helminths and arthropods (such as mites and cockroaches) antigens. A proportion of low-titer IgE antibodies detected by the IgE assay used in our study could be low-affinity IgE antibodies to helminth antigens that cross-react with arthropod allergens. Such low titer IgE may be less effective in interacting with allergens to activate mast cells [25,28,29]. In support, our data showed that children with negative SPTs had lower levels of sIgE to *B*. *tropicalis* than children SPT positives. It is possible that the sIgE threshold for SPT reactivity is higher than the assay cut-off. Although very low specific IgE levels have been associated with anaphylaxis to venom allergens [30] this may not be true for aeroallergens such as mite.

There is growing evidence for significant IgE cross-reactivity between helminths and a wide range of invertebrate allergens [31,32]. A previous observation from our research group showed that the absorption of sera with an extract of *A*. *lumbricoides* antigens decreased levels of sIgE to *B*. *tropicalis*[16]. An alternative hypothesis to explain the role played by helminths in the down-modulation of SPT reactivity is the enhancement of Treg cells and regulatory cytokines production, such as IL-10 and TGF-β, that may inhibit mast cell degranulation [24,29,31]. Although regulatory cytokines can theoretically also down-modulate IgE production [33,34], they likely do not do this as efficiently as inhibition of the effector phase of the type I hypersensitivity response in skin. In fact helminths are strong inducers of total and parasite-specific IgE despite the high levels of IL-10 produced during chronic helminth infections [13].

We investigated the effect of markers of viral infections (i.e. Hepatitis A, *Herpes simplex*, *Herpes zoster* and Epstein-Barr viruses) on SPT reactivity. The only association observed was a reduced SPT reactivity to *D*. *pteronyssinus* in sIgE positive children among those with IgG to *H*. *simplex* virus. The association of infections with reduced SPT reactivity could be explained by the induction of antigen-specific regulatory responses by pathogens that share sequence homology with allergens, but also with non-specific suppression of the immune response.

The finding of low biologic activity of IgE cross-reactive to carbohydrate epitopes of glycoproteins has been reported in grass pollen sensitized patients who had specific IgE to peanut but no skin sensitivity to peanut [7]. The authors conclude that ‘non-effector’ IgE contributed to the discrepancy between sIgE and SPT. For this reason, we investigated also if carbohydrate determinants of *B*. *tropicalis* allergens could play a role in the discrepancy between sIgE and SPT. We observed that carbohydrate depletion of *B*. *tropicalis* extracts led to a significant reduction in sIgE levels among children who were sIgE+/SPT- to *B*. *tropicalis*. Although the glycoproteins in this case were from *B*. *tropicali*s, the same is likely to be true for carbohydrate molecules present in helminth extracts for which cross-reactive IgE responses have been reported with dust mites [35].

Although helminths are important inducers of polyclonal IgE as we [27] and others [2, 3, 4] have shown, our data provided only limited evidence for high levels of policlonal IgE explaining the dissociation between sIgE and SPT. There was some evidence that the SPT-sIgE association was weaker among children with the highest total IgE levels compared with children with ‘moderate’ or ‘low’ levels, although a statistically significant interaction was seen only for the former comparison (S2 Table). Previous study showed that total IgE may be contribute to the dissociation between sIgE and SPT observed in some populations [1,36].

Another possible explanation for absence of SPT reactivity in the presence of specific IgE for the same allergen could be host genetic factors. Previous studies linked polymorphisms on *FCER1A* with a reduction in sIgE levels and SPT reactivity [8]. However, none of these studies were done in a sIgE-positive population perhaps explaining why we did not observe relationships between SNPs on *FCER1A* and SPT reactivity here. Alternatively, one might think that SNPs on Immunoglobulin Heavy Constant Epsilon gene (*IGHE*; located at 14q32) could also play a role in SPT reactivity. In previous studies the genes most consistently shown to have effects included *IL4*, *IL13*, *IL4RA* and *FCERB1* and more recently *IL33* and *ST2* and the *IGH* genes, both *IGHG* and *IGHE* [37,38]. In the present study, we have described for the first time a negative association between rs61737468 on *IGHE* and SPT reactivity. We could speculate that changes in the IgE heavy chain could decrease its affinity for FCɛRI and interfere with mast cell degranulation in the skin. Further molecular studies are required in other populations to elucidate how rs61737468 might affect SPT reactivity.

## Conclusions

In conclusion, we have explored the role of poor hygiene exposures and genetic factors as an explanation for reduced SPT positivity observed in some LMIC settings in children with specific IgE for the same aeroallergens. Our observations do provide support for a role of poor hygiene exposures including childhood infections in mediating this effect, but other factors may have a role including host genetic factors, indicating that diverse mechanisms could be important. Potential mediating mechanisms identified here were increased glycosylation of aeroallergen-specific IgE and high levels of total IgE. A better understanding of how allergic effector responses are modulated could lead to the development of novel strategies for the prevention and control of allergic diseases.

## Supporting information

S1 Table Crude and adjusted odds-ratios (OR) of the associations without statistical significance, between possible determinants and skin prick test (SPT) least one allergen, *B. tropicalis* or *D. pteronyssinus* among 491 children with specific serum IgE for the respective allergen. *Adjusted for the *a priori* confounders (age, gender and parental asthma); **Ascaris lumbricoides and *Trichuris trichiura*
(DOCX)

S2 Table Effect of total IgE serum levels in the association of sIgE with SPT reactivity in 1,353 studied children. p-valor (Breslow-Day). OR: 1^st^ with 2^nd^ tertile p = 0,531; 1^st^ with 3^rd^ tertile p = 0,174 and 2^nd^ with 3^rd^ tertile = 0,040.
(DOCX)

## Abbreviations

Bla g 2: recombinant allergen 2 from *Blatella germanica*
Blo t 5: recombinant allergen 5 from *Blomia tropicalis*
BMI: body mass index
Can f 1: recombinant allergen 1 from *Canis familiaris*
Der p 1: recombinant allergen 1 from *Dermatophagoides pteronysinus*
EBV: Epstein-Baar virus
FcɛRI: High affinity IgE receptor
FCER1A: FcɛRI gene
Fel d 1: recombinant allergen 1 from *Felis domesticus*
GNI: gross national income
HAV: Hepatitis A virus
HSV: Herpes simplex virus
HZV: Herpes zoster virus
ISAAC: International Study of Asthma and Allergies in Childhood
IgE: Immunoglobulin E antibody
IGHE: IgE gene
IgG: Immunoglobulin G antibody
kU/L: kilounits per liter
SCAALA: Social Change, Asthma and Allergy in Latin America
sIgE: aeroallergen-specific immunoglobulin E antibody
SNP: single nucleotide polymorphisms
SPT: skin prick test
Th1: T helper 1
Th2: T helper 2
