# Supplementary material for: Dissociation between skin test reactivity and anti-aeroallergen IgE: Determinants among urban Brazilian children
Source: PLoS One. 2017 Mar 28;12(3):e0174089. doi: 10.1371/journal.pone.0174089 (PMC5369757; doi:10.1371/journal.pone.0174089)
Supplement: S1 Table — *Adjusted for the a priori confounders (age, gender and parental asthma); **Ascaris lumbricoides and Trichuris trichiura (DOCX) [file pone.0174089.s001.docx]

**Table S1. Crude and adjusted odds-ratios (OR) of the associations without statistical significance, between possible determinants and skin prick test (SPT) least one allergen,**

***B. tropicalis* or *D. pteronyssinus* among 491 children with specific serum IgE for the respective allergen**

| **Possible determinants** | **Positive SPT for at least one allergen in children with specific IgE fpr the same allergen** | | |
| --- | --- | --- | --- |
|  | N(%) | Crude OR  (95%CI) | *Adjusted OR  (95% CI) |
| **Cockroaches in household** |  |  |  |
| No | 74(69.8) | 1 | 1 |
| Yes | 247(64.3) | 0.78[0.49-1.24] | 0.74[0.46-1.19] |
| **Sewage system at home** |  |  |  |
| **HAV** | 129(64.2) | 1 | 1 |
| No | 117(71.3) | 1.38[0.89-2.17] | 1.27[0.80-2.01] |
| Yes | 65(59.6) | 0.82[0.51-1.33] | 0.73[0.44-1.20] |
| **VZV** |  |  |  |
| No | 270(66.5) | 1 | 1 |
| Yes | 50(60.2) | 0.76[0.47-1.24] | 0.74[0.45-1.21] |
| ***T.gondii*** |  |  |  |
| No | 178(65.9) | 1 | 1 |
| Yes | 142(65.4) | 0.98[0.67-1.43] | 0.89[0.61-1.32] |
| ***H. pilory*** |  |  |  |
| No | 276(67.3) | 1 | 1 |
| Yes | 44(58.7) | 0.69[0.42-1.14] | 0.66[0.40-1.10] |
| **Total IgE** |  |  |  |
| 1° tertile | 224(67.5) | 1 | 1 |
| 2° tertile | 78(60.5) | 0.74[0.48-1.12] | 0.73[0.48-1.13] |
| 3° tertile |  |  |  |
| **Possible determinants** | | **Positive SPT to *B. tropicalis* in children with specific IgE for this allergen** | |
| **Cockroaches in household** |  |  |  |
| No | 61(62.9) | 1 | 1 |
| Yes | 188(54.0) | 0.69[0.44-1.10] | 0.69[0.43-1.10] |
| **% of neighborhood covered with daily garbage collection** |  |  |  |
| >= 66% | 88(62.0) | 1 | 1 |
| 33% - 66% | 28(57.1) | 0.82[0.42-1.58] | 0.81[0.42-1.57] |
| 0 – 33% | 133(52.2) | 0.67[0.44-1.02] | 0.68[0.45-1.04] |
| **Sewage system at home** |  |  |  |
| ≥ %57 | 95(50.3) | 1 | 1 |
| 50% \|--\| 75% | 90(60.4) | 1.51[0.98-2.33] | 1.43[0.92-2.24] |
| 0%\|--\| 50% | 55(59.8) | 1.47[0.89-2.44] | 1.38[0.82-2.32] |
| **Epstein-Barr virus** |  |  |  |
| No | 42(66.7) | 1 | 1 |
| Yes | 206(54.2) | 0.59[0.34-1.04] | 0.61[0.34-1.07] |
| **Herpes simplex virus** |  |  |  |
| No | 133(60.2) | 1 | 1 |
| Yes | 116(51.6) | 0.70[0.48-1.02] | 0.68[0.47-1.01] |
| **HAV** |  |  |  |
| No | 213(57.3) | 1 | 1 |
| Yes | 34(47.2) | 0.67[0.40-1.11] | 0.65[0.39-1.08] |
| **VZV** |  |  |  |
| No | 129(52.9) | 1 | 1 |
| Yes | 118(59.6) | 1.31[0.90-1.92] | 1.25[0.85-1.84] |
| ***T. gondii*** |  |  |  |
| No | 212(56.4) | 1 | 1 |
| Yes | 35(53.8) | 0.90[0.53-1.53] | 0.88[0.52-1.50] |
| ***H. pilory*** |  |  |  |
| No | 1.76(57.3) | 1 | 1 |
| Yes | 61(54.0) | 0.87[0.57-1.35] | 0.86[0.55-1.33] |
| **Total IgE** |  |  |  |
| 1° tertile | 88 (61.1) | 1 | 1 |
| 2° tertile | 75 (51.0) | 0.66 [0.42-1.06] | 0.65 [0.41-1.04] |
| 3° tertile | 86 (55.5) | 0.79 [0.50-1.26] | 0.76 [0.48-1.22] |
| **Possible determinants** | | **Positive SPT to *D. pteronyssinus* in children with specific IgE for this allergen** | |
| **Number of siblings** |  |  |  |
| 0-1 | 104(62.3) | 1 | 1 |
| >=2 | 70(59.3) | 0.88[0.54-1.43] | 0.85[0.52-1.39] |
| **Rodent infestation in household** |  |  |  |
| No | 84(63.2) | 1 | 1 |
| Yes | 89(58.9) | 0.84[0.52-1.35] | 0.77[0.47-1.26] |
| **Cat in household** |  |  |  |
| **No** | 141(59.0) | 1 | 1 |
| Yes | 33(71.7) | 1.76[0.88-3.52] | 1.76[0.87-3.54] |
| **Cockroaches in household** |  |  |  |
| No | 44(65.7) | 1 | 1 |
| Yes | 129(59.4) | 0.77[0.43-1.36] | 0.75[0.42-1.35] |
| **Sewage system at home** |  |  |  |
| ≥ %57 | 69(58.0) | 1 | 1 |
| 50% \|--\| 75% | 65(55.2) | 1.57[0.89-2.76] | 1.56[0.86-2.82] |
| 0%\|--\| 50% | 32(55.2) | 0.89[0.47-1.68] | 0.87[0.45-1.69] |
| **** Intestinal helminth infection** |  |  |  |
| No | 145(62.2) | 1 | 1 |
| Yes | 28(56.0) | 0.77[0.42-1.43] | 0.73[0.39-1.38] |
| **Epstein-Barr virus** |  |  |  |
| No | 30(68.2) | 1 | 1 |
| Yes | 142(59.4) | 0.68[0.34-1.35] | 0.70[0.35-1.39] |
| **HAV** |  |  |  |
| No | 144(60.5) | 1 | 1 |
| Yes | 29(64.4) | 1.18[0.61-2.30] | 1.23[0.63-2.41] |
| **VZV** |  |  |  |
| No | 94(59.1) | 1 | 1 |
| Yes | 78(63.4) | 1.20[0.74-1.94] | 1.19[0.72-1.95] |
| ***T. gondii*** |  |  |  |
| No | 153(63.0) | 1 | 1 |
| Yes | 20(52.6) | 0.65[0.33-1.30] | 0.63[0.31-1.27] |
| ***H. pilory*** |  |  |  |
| No | 122(62.2) | 1 | 1 |
| Yes | 43(60.6) | 0.93[0.53-1.62] | 0.92[0.52-1.61] |
| **Total IgE** |  |  |  |
| 1° tertile | 54 (65.1) | 1 | 1 |
| 2° tertile | 53 (60.9) | 0.84 [0.45-1.56] | 0.78 [0.41-1.47] |
| 3° tertile | 67 (58.3) | 0.75 [0.42-1.34] | 0.66 [0.36-1.20] |

*Adjusted for the *a* *priori* confounders (age, gender and parental asthma); ***Ascaris lumbricoides* and *Trichuris trichiura.*
