# Supplementary material for: Dissociation between skin test reactivity and anti-aeroallergen IgE: Determinants among urban Brazilian children
Source: PLoS One. 2017 Mar 28;12(3):e0174089. doi: 10.1371/journal.pone.0174089 (PMC5369757; doi:10.1371/journal.pone.0174089)
Supplement: S2 Table — p-valor (Breslow-Day). OR: 1st with 2nd tertile p = 0,531; 1st with 3rd tertile p = 0,174 and 2nd with 3rd tertile = 0,040. (DOCX) [file pone.0174089.s002.docx]

**Table S2. Effect of total IgE serum levels in the association of sIgE with SPT reactivity in 1,353 studied children**

| **N = 1353** | **Total IgE 1° tertile** | | **Total IgE 2° tertile** | | **Total IgE 3° tertile** | |
| --- | --- | --- | --- | --- | --- | --- |
|  | **SPT** | | **SPT** | | **SPT** | |
| **sIgE** | n(%)/N | Crude OR  (95% CI) | n(%)/N | Crude OR  (95% CI) | n(%)/N | Crude OR  (95% CI) |
| Neg | 25 (7.0)/357 | 1 | 25 (7.7)/323 | 1 | 22 (13.3)/165 | 1 |
| Pos | 58 (61.1)/95 | 20.82  [11.67-37.14] | 88 (69.3)/127 | 26.90  [15.43-46.88] | 187 (65.4)/286 | 12.28  [7.37-20.46] |

p-valor (Breslow-Day).  OR:   1^st^ with 2^nd^tertile  p= 0,531;  1^st^ with 3^rd^ tertile p= 0,174 and 2^nd^ with 3^rd^ tertile = 0,040.
